# Evaluation of the envision endoscopy SimpleStitch suturing system for closure of gastrointestinal defects in a porcine model

**DOI:** 10.1007/s00464-026-12718-4

**Published:** 2026-03-10

**Authors:** Manik Aggarwal, Jad P. AbiMansour, Reem Matar, Manus Rugivarodom, Yara Salameh, Hadi Abou Zeid, Shunsuke Kamba, Naomi M. Gades, Elizabeth Rajan, Andrew C. Storm

**Affiliations:** 1 Division of Gastroenterology and Hepatology, Mayo Clinic, Rochester, MN USA; 2 Developmental Endoscopy Unit, Mayo Clinic, Rochester, MN USA; 3 Division of Gastroenterology, Wake Forest University School of Medicine, Winston-Salem, NC USA

**Keywords:** Endoscopic suturing, Resection, Porcine, Defect closure

## Abstract

**Background:**

Flexible endoscopic suturing tools are complex and may have a long learning curve. This porcine study evaluated the safety and performance of a simplified suturing system compared with a commercially available device for the repair of gastrointestinal mucosal defects.

**Methods:**

This IACUC-approved study included four healthy swine. A total of ten defects (six in the stomach and four in the rectosigmoid colon) were created in each animal. Defects were randomly assigned to closure with either the novel or the commercially available system using a therapeutic gastroscope. Technical success was defined as mucosal closure of the defect with the inability to visualize any significant portion of the resection bed. Additional performance metrics included procedure time and device ease of use (assessed using the NASA Task Load Index [TLI]) and adverse events.

**Results:**

No adverse events were reported post-procedurally for any of the test animals. The proportion of target resection sites achieving technical success was 100% in both treatment groups. The mean SimpleStitch NASA-TLI score was lower compared to the OverStitch device. Closure times were similar between the two devices. Histological assessment scores indicated expected healing response without evidence of perforation, leakage, or abscess formation.

**Conclusion:**

A novel full-thickness suturing system safely and effectively closed mucosal defects. Lower NASA-TLI scores suggest that the novel suturing device may offer simpler, less demanding use compared to the predicate device, potentially reducing the learning curve for endoscopic suturing procedures.

**Supplementary Information:**

The online version contains supplementary material available at 10.1007/s00464-026-12718-4.

Endoscopic procedures for gastrointestinal (GI) tract lesions, including mucosal, submucosal, and full-thickness resections, are increasingly adopted in clinical practice. The increasing use of endoscopic resection has driven demand for effective closure methods to reduce complications and aid healing. Endoscopic resection, particularly of larger (> 2 cm) lesions, is associated with potential adverse events, most notably perforation (up to 3% of cases) and bleeding in up to 10% of cases [1]. Prophylactic closure reduces these risks, with several closure devices available depending on defect characteristics [2].

Endoscopic suturing systems developed over the last two decades have transformed defect closure, particularly after resection of large lesions, fistulas, and leaks. The predicate 2nd generation OverStitch system (Boston Scientific, Marlborough, MA) has high technical and clinical success [3]. However, the system is constrained by the need for a dual-channel endoscope with a tower design that may limit visualization and subsequent difficulty in maneuvering, particularly when placing purse-string and other complex patterned sutures. Furthermore, this predicate suturing system requires multiple steps for each suture placement, risking device failure with any single misstep [4].

To address these limitations, a novel suturing system (SimpleStitch, Envision Endoscopy, Somerville, MA) was developed. This compact, intuitive device attaches to a single-channel endoscope, potentially simplifying closure. A circular notched needle with a cable-driven mechanism facilitates continuous or interrupted suturing with improved visualization. The primary objective of this study was to evaluate the safety and performance of the SimpleStitch system in comparison to the predicate OverStitch device.

## Materials and methods

### Study design and animal model

The study was conducted in a porcine model to simulate clinical conditions of gastric and rectosigmoid mucosal defect closure. The study was conducted in accordance with the *Guide for the Care and Use of Laboratory Animals: Eighth Edition* and approved by the Mayo Clinic Institutional Animal Care and Use Committee (IACUC) in compliance with AAALAC® International accreditation, Office of Laboratory Animal Welfare (OLAW) assurance, and United States Department of Agriculture (USDA) registration. Four healthy domestic pigs (50–60 kg) were selected due to the anatomical and physiological similarity of porcine GI tissues to human tissues. Before study assignment, the animals were verified to be free of any disease/condition that might reasonably be expected to interfere with the study as determined by a veterinarian. This study was conducted following ARRIVE 2.0 guidelines for animal preclinical studies.

### Defect creation and randomization

**Fig. 1:**
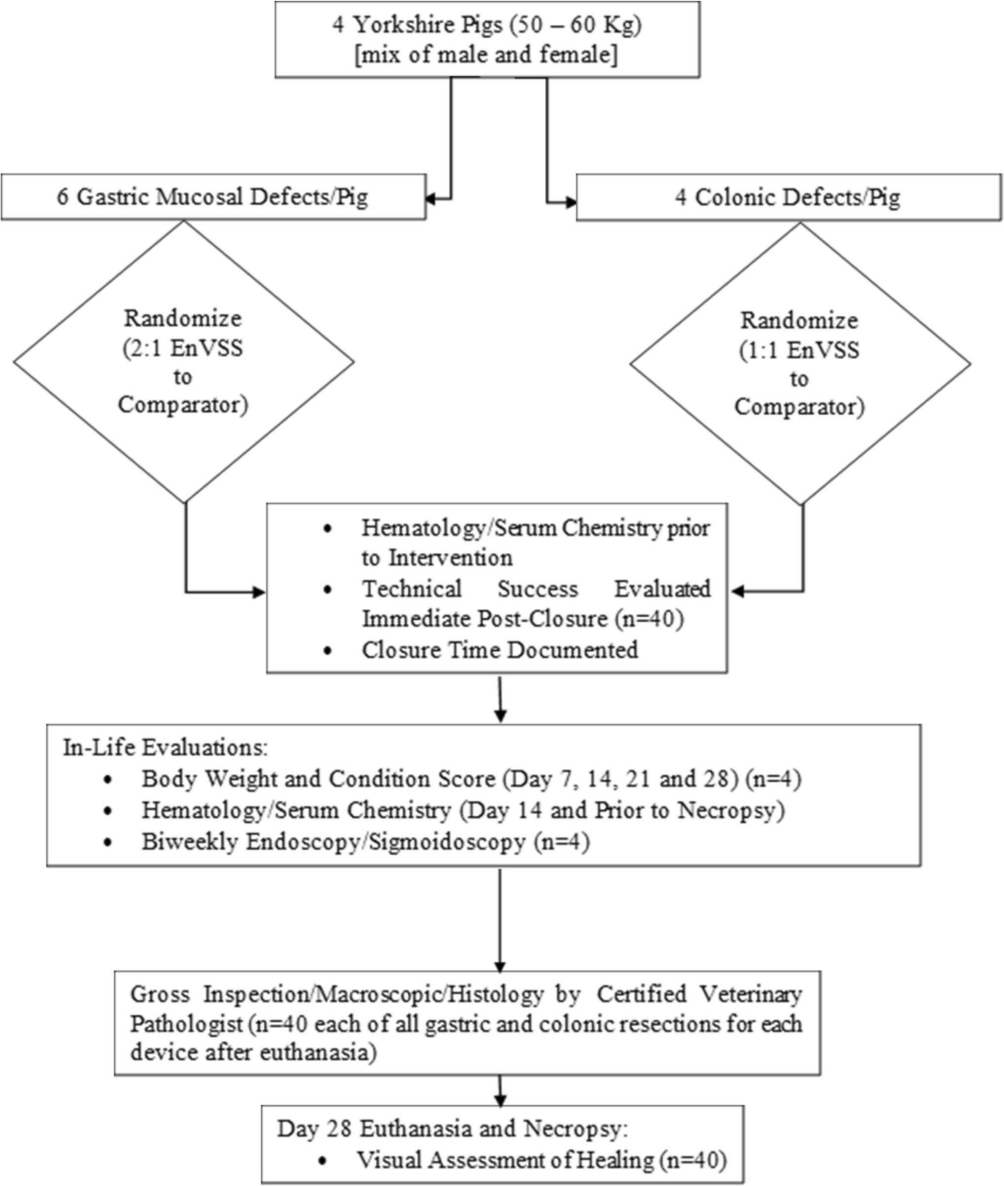
Study overview

A schematic representation of the study is depicted in Fig. 1. On Day 0, animals underwent anesthesia, upper endoscopy (GIF-H180 single channel, Olympus America, Center Valley, Pennsylvania, United States) to ensure normal mucosa and anatomy. Defects were created using mucosal resections, performed using a hybrid technique involving submucosal injection (sterile saline, 1:100,000 epinephrine, and methylene blue) with a goal range for a 2–4 cm diameter cushion followed by circumferential electrosurgical needle knife (Olympus America, Center Valley, Pennsylvania, USA) excision of the mucosa. As needed, hybrid EMR (endoscopic mucosal resection) using a Lariat snare (Steris, Mentor, OH, USA) was performed to excise either in a single or piecemeal resection. Defects were measured using a snare with centimeter markings to ensure resections were at least 2–4 cm.

A total of four animals were used in this study. Each animal underwent endoscopic creation of 10 mucosal defects, totaling 40 defects across the study. In each animal, six mucosal resections were performed in the stomach and four resections in the rectosigmoid colon. Prescribed gastric lesion sites were as follows: anterior and posterior fundus (2), anterior and posterior body (2), and anterior and posterior antrum (2). In the rectosigmoid colon, lesion sites were as follows: anterior and posterior colon (2), anterior and posterior rectum (2). None of the wounds were left open or untreated. As this was a pilot feasibility study, no formal power calculation was performed.

Defects in each animal were randomized for closure with either the test or the control system prior to study initiation. Four gastric sites were closed with the test device and two with the control device. The rectosigmoid defects were allocated in a 1:1 fashion to the test and control device. Randomization assigned four gastric sites to the test device and two to the control; rectosigmoid closures were 1:1 (Supplementary Table 1).

### Devices

**Fig. 2:**
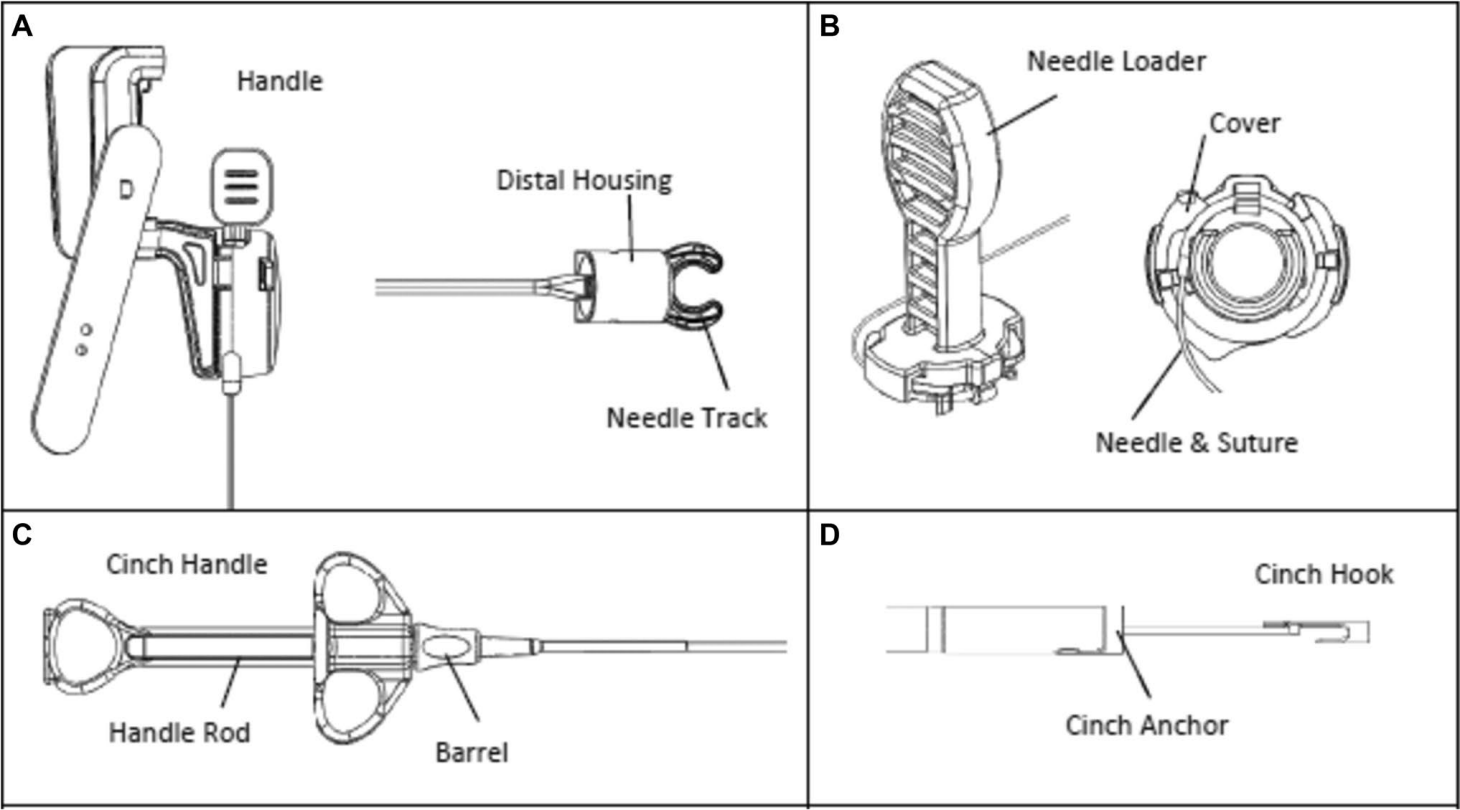
Schematic representation of components of SimpleStitch suturing system. **A** Handle is placed over the biopsy cap on to the endoscope and is secured with a strap. The distal housing slides onto the distal end of the endoscope which is flush with the housing to maximize visualization. The housing is tightened around the endoscope using a screwdriver provided in the packaging. **B** The needle loader attaches to the distal housing and the safety spacer is then removed to complete the assembly. **C** The cinch handle to actuate firing of a suture cinch. **D** The cinch hook is brought back into the anchor portion to secure the final suture construct

**Fig. 3:**
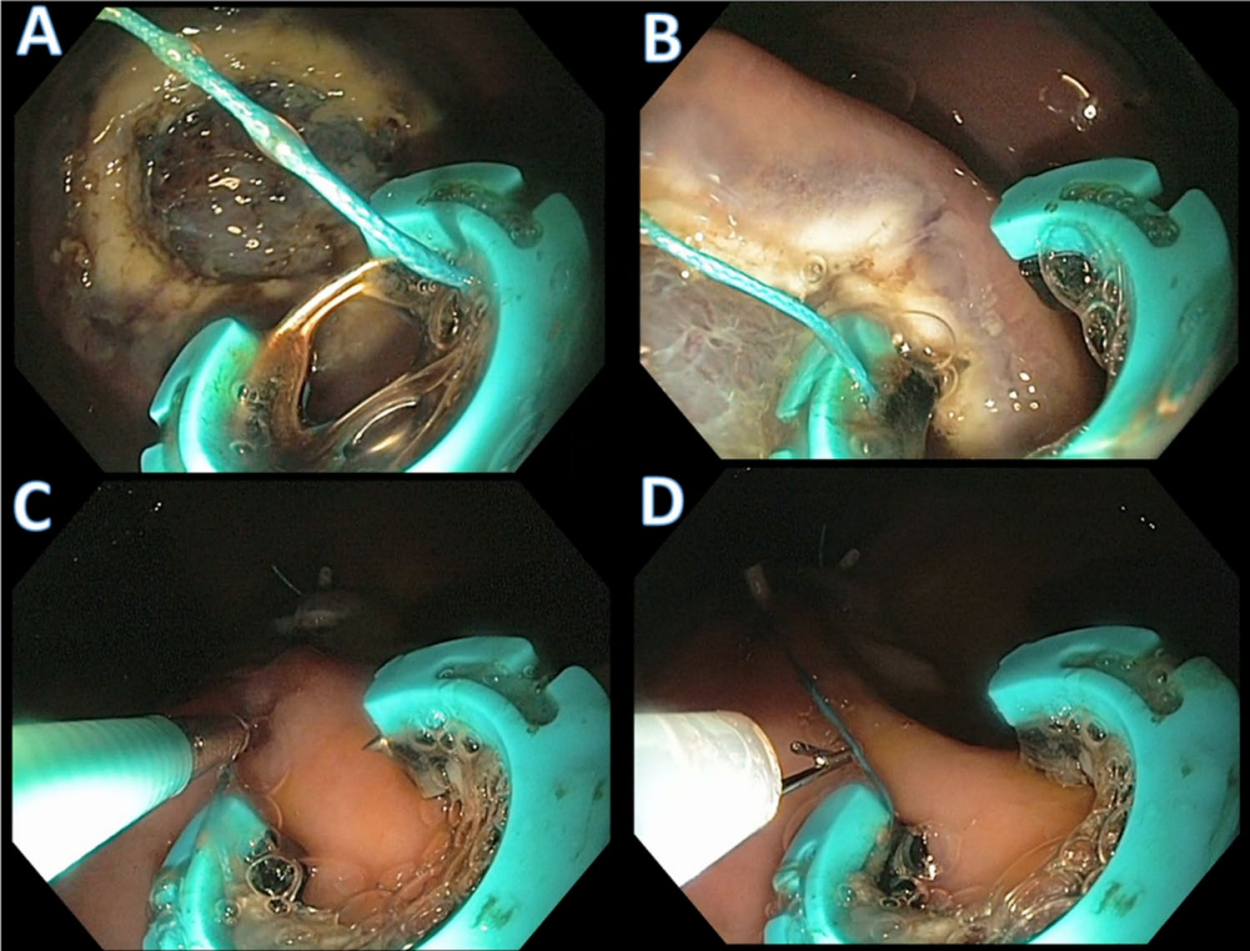
Representative images of mucosal defect closure with the novel SimpleStitch suturing system. **A** Circular suturing device in closed position with a 15 mm mucosal defect. **B** Tissue ‘bite’, the circular design allows for capturing full-thickness tissue bites. **C** Through-the-scope tissue cinch is used to tighten and secure the suture construct. **D** Novel through-the-scope suture cutting tool used to cut the suture after firing the cinch

The test arm employed the SimpleStitch Suturing System, a recent FDA-cleared flexible endoscopic suturing device anticipated to soon be clinically available. This system comprises a single-channel endoscope suturing device, cinch delivery catheter, and suture hook cutter (Fig. 2). The suturing device attaches to the endoscope, and the cinching device is placed through the endoscope’s working channel (Fig. 3). A simple endoscopic suture cutter developed for the suturing system is used to cut the suture instead of traditional endoscopic scissors or loop cutter. Like the cinching device, the cutting device is passed through the working channel of the endoscope (Fig. 2). Device assembly and attachment to the endoscope are depicted in video-1.

The control device was an FDA-cleared and commercially available full-thickness suturing device (OverStitch, Boston Scientific, Marlborough, MA) mounted on a dual-channel therapeutic endoscope. The device components include: (1) a metal end cap; (2) a curved suture arm (needle driver); (3) an anchor exchange (pickup) catheter; (4) a tissue anchor or T-tag (needle); and a (5) cinching device, which is used to secure and cut the suture.

A dual-channel gastroscope (GIF-2TH160, Olympus) was used for OverStitch procedures, while a standard single-channel gastroscope (GIF-H180) was used for SimpleStitch. Closures were performed by two experienced endoscopists (JPA and ACS, each with > 3 years’ suturing experience).

### In-life evaluations

Animals were monitored post-procedure for signs of distress or adverse effects. Body weights and body condition scores (BCS) were recorded at baseline and weekly. Endoscopic assessments were performed on Day 14 and Day 28 to evaluate healing progression using the Mayo Clinic modified Forrest classification [5]. Blood samples were collected on Days 0, 14, and 28 for hematology and serum chemistry analysis.

### Necropsy and histology

On Day 28, animals were euthanized for necropsy. Macroscopic inspection of closure sites was performed to determine residual defect, tissue integrity, and adverse reactions. Histological evaluation was conducted on all resection sites to determine microscopic inflammatory responses and healing progression. Ten (10) ml of whole blood/pig was collected via the femoral vein and split between a Lithium Heparin tube and an Ethylenediaminetetraacetic acid (EDTA) tube prior to the start of the resection and endoscopy procedures for chemistry and a complete blood count, respectively. An Abaxis Piccolo Xpress was used to provide chemistry results, and an Abaxis HM5 Analyzer was used to generate the complete blood count.

At necropsy, tissues for histopathology were collected, fixed in 10% neutral buffered formalin, and sent to the Mayo Clinic Research Histology Core. Devices were removed, and sites were trimmed. Samples underwent standard processing, sectioned at 5 µm, and stained with hematoxylin and eosin or Masson’s trichrome. An American College of Veterinary Pathology (ACVP) board-certified veterinary pathologist evaluated sections using a 5-point semi-quantitative scale: 0 (within normal limits) to 4 (marked changes).

### Study outcomes

1. Technical success: This was defined as mucosal closure of the defect with inability to visualize any significant portion of the resection bed. This was ascertained after each defect closure
2. Procedure time: This was calculated from the initiation of the first tissue “bite” taken through cinch deployment and suture cutting.
3. Ease of use: At the completion of each study subject’s initial (Day 0) procedure, endoscopists rated device preparation/setup (specifically ability to load needle/suture into device/attach to scope and insert endoscope into an animal) using a 5-point Likert difficulty scale (where 0 = inability to close defect, 1 = very difficult/unsatisfactory, 3 = medium/neutral and 5 = very easy/very satisfactory); and to evaluate overall device usability using the National Aeronautics and Space Administration Task Load Index (NASA-TLI). The NASA-TLI is a multi-dimensional rating procedure that provides an overall workload score based on a weighted average of ratings on six subscales: Mental Demands, Physical Demands, Temporal Demands, Own Performance, Effort, and Frustration. With NASA Task Load Index higher scores are associated with a higher demand in overall usability and workload [6]. This index has been previously validated to assess workload during flexible endoscopic procedures [7].

### Statistical analysis

A descriptive analysis using medians and range for all variables was reported collectively and separately for stomach and colon. Data were analyzed in JMP version 14 (SAS Institute Inc., Cary, NC) using parametric and non-parametric tests. Comparisons of diameter of residual defect, closure time, and number of residual suture/cinch closure constructs during follow-up were made. NASA Task Load Index was performed to determine user workload of each device [10].

## Results

In total, 40 mucosal defects were created (10 defects per animal), 24 gastric and 16 colonic. Sixteen gastric sites were closed with the test system and eight with the control. Mean gastric defect diameters were similar between the groups (test: 31.5 ± 6.4 mm; control: 26.5 ± 3.6 mm) (Table 1). Colon resection diameters were also similar between the two groups (26.7 ± 3.6 mm for the test arm and 26.4 ± 5.1 mm for the predicate arm). Technical success, defined as complete mucosal closure with no residual visualization of the resection bed, was achieved in 100% of defects for both the test and predicate systems.

**Table 1:** Comparison of novel test (SimpleStitch) and control (OverStitch) suturing systems for closure of gastrointestinal defects. Data are presented as mean ± standard deviation (SD)

Variable	Gastric defects		Colonic defects	
	SimpleStitch (*n* = 16)	OverStitch (*n* = 8)	SimpleStitch (*n* = 8)	OverStitch (*n* = 8)
Induced lesion diameter (mm)	31.5 ± 6.4	26.5 ± 3.6	26.7 ± 3.6	26.4 ± 5.1
Follow up (day 14) diameter (mm)	10.4 ± 4.1	11.6 ± 6.2	8.8 ± 8.9	10.5 ± 6.2
Follow up final (day 28) diameter (mm)	0.0 ± 0.0	0.0 ± 0.0	0.0 ± 0.0	0.0 ± 0.0
Healing at follow up (day 14) III	100%	100%	100%	100%
Healing at necropsy (day 28) IV	100%	100%	100%	100%
No suture retained in final follow up	68%	62.5%	75%	62.5%

Gastric mucosal closure times with test system (6:09 min [range 2:20–13:15]) were similar when compared with the control device (4:43 min [range 2:43–8:10]). Rectosigmoid closure times were also comparable between the test (5:31 min [range 3:22–11:50]) and control systems (4 min [range 2:43–5:50]). A similar number of suture bites were needed with each system for defect closure (4–5 suture bites per defect with both systems). A representative closure using the test system is depicted in video 2.

In one animal (Pig no. 2), two full-thickness perforations (approximately 5–8 mm) occurred inadvertently during supplemental snare excisions of the colon mucosectomy margins. Despite the perforations occurring, complete closure was successful for both, one performed with each device: the anterior perforation site was repaired with the predicate system, and the posterior perforation site was closed using the test system. Post-closure assessment confirmed no significant pneumoperitoneum, and the animal remained clinically stable without signs of peritonitis or distress throughout the 28-day follow-up period.

### Ease of use and NASA task load index

Device usability was evaluated using a 5-point Likert scale and the NASA-TLI. Both systems were rated 5/5 (very easy/very satisfactory) across all study animals across device preparation, loading and attachment to the endoscope. The median SimpleStitch NASA-TLI was 17.5 (10–25) and OverStitch: 31.5 (25–43) (*p* = 0.01) (Table 2). Notably, SimpleStitch rated lower than or equal to the OverStitch device in demand/task load in all subcomponents of the NASA-TLI (Supplementary Table 2).

**Table 2:** NASA task load Index scores for the simplestitch (SS) and overstitch (OS) devices

	Pig 1		Pig 2		Pig 3		Pig 4	
	SS	OS	SS	OS	SS	OS	SS	OS
Device preparation and setup (0–5) 0 = inability to perform task, 1 = very difficult/unsatisfactory, 3 = medium/neutral, 5 = very easy/very satisfactory	5	5	5	5	5	5	5	5
Overall usability NASA task load index								
Mental demand (0–20)	8	12	4	6	3	4	2	3
Physical demand (0–20)	0	5	4	7	3	4	2	7
Temporal demand (0–20)	4	5	3	5	4	5	2	2
Performance (0–20)	0	3	5	6	2	4	0	2
Effort (0–20)	2	10	4	6	2	6	2	7
Frustration (0–20)	4	8	5	5	3	5	2	4
Total score	18	43	25	35	17	28	10	25
SS overall median score (Range)	17.5 (10–25)							
OS overall median score (Range)	31.5 (25–43)							

### Healing progression

Healing progression was assessed endoscopically on Day 14 and Day 28 using the Mayo Developmental Endoscopy Unit Score [5] for resection bed healing (Supplementary Table 2). At Day 14, all closure sites exhibited Grade III healing (clean-based ulcers) without bleeding, residual defects, or stigmata. At two weeks, there was a similar concentric reduction in all sites that was comparable between the test (mean reduction in diameter Δ 10.4 ± 4.1 mm) and the control systems (Δ 11.3 ± 6.2 mm). By Day 28, all sites achieved Grade IV healing, defined as complete mucosal closure with no remaining ulceration or defect.

### Histology and necropsy observations

At necropsy, macroscopic examination revealed no abnormalities in the abdominal or thoracic cavities. All closure sites were fully healed, with no evidence of perforation, abscess formation, or tissue necrosis. The opened stomachs revealed healed resection sites with retained suture constructs in a similar number of cases between the two systems (31.2% vs. 37.5% with the test and predicate systems, respectively). The opened rectosigmoid segments of the colon revealed healed resection sites with retained suture constructs in 5 cases (25% vs. 37.5% with the test and predicate systems, respectively).

**Fig. 4:**
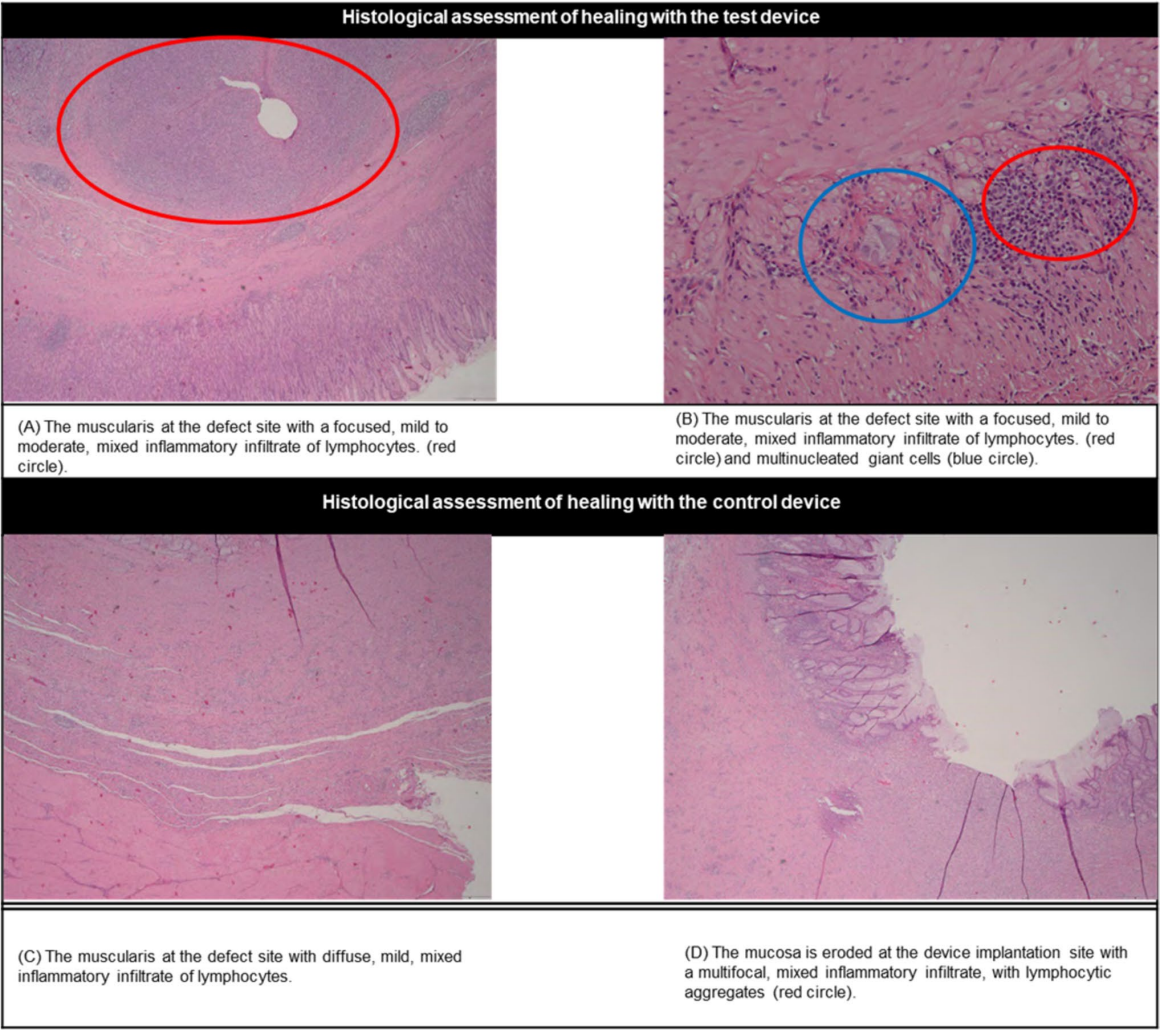
(Left) Hematoxylin and Eosin (H&E)-stained section of muscularis at the defect site (well healed) with a focused, mild to moderate, mixed inflammatory infiltrate of lymphocytes. (Right) Hematoxylin and Eosin-stained section of muscularis at the defect site (well healed) with a focused, mild to moderate, mixed inflammatory infiltrate of lymphocytes (red circle) and multinucleated giant cells (blue circle) (Color figure online)

Histology showed no significant differences in the inflammation and tissue reaction scores between the two suturing systems (Fig. 4; Supplementary Table 3). Inflammation was primarily composed of mixed lymphocytic infiltrates, indicative of normal tissue healing. Fibrin, thrombi, granulation tissue, necrosis, hemorrhage, hemosiderin, edema, and mineralization were not evident at any of the treatment sites regardless of device. There was no evidence of perforation/leakage, bacterial infection, severe hemorrhage, or abscess formation in any of the evaluated samples.

### Adverse events

No adverse events occurred during follow-up. All animals demonstrated progressive weight gain and achieved a body condition score of 4 (fat) by Day 28, consistent with the safety and tolerability of both devices.

## Discussion

Endoscopic suturing has transformed defect closure and tissue remodeling during flexible endoscopic interventions, enabling safe resection of large pre-cancerous and cancerous lesions. This pre-clinical study evaluated a novel single-channel endoscopic suturing system (SimpleStitch) with a circular suturing arm designed to mimic laparoscopic suturing. The study device successfully closed all defects, including inadvertent full-thickness perforations, with comparable closure times to the predicate device despite the users, experts with the control device, having limited experience with the test device. NASA-TLI scores were significantly lower for the test device, indicating reduced workloads and easier use. The findings demonstrate that the test suturing system is safe, efficient, and an easy-to-use alternative to the predicate dual-channel device for large and complicated defect closure.

The SimpleStitch system successfully closed all defects with comparable rates of healing (at 2 and 4 weeks) when compared with the test platform. Based on endoscopic feedback, the novel device was easy to deploy in the narrow caliber porcine colon suggesting this system can be employed throughout the human intestine. Closure times with both systems were comparable despite endoscopists having no prior experience with the novel system and were comparable to closure times with endoscopic suturing systems in prior studies [8, 9]. Histological findings confirmed minimal tissue reaction and favorable healing, consistent with durable closure. All inflammation and tissue reactions observed histologically in this study were interpreted as part of the expected healing process, were modest (i.e., did not affect the overall integrity and viability of the gastrointestinal wall) and similar between the devices.

The device was easy to set up and endoscopists found the device to have lower workload as evidenced by the significantly lower NASA-TLI scores. Particularly, the physical demand and the effort components were scored to be lower for the test system when compared to the predicate platform. The intuitive mechanism of the test system, which reduces the number of steps needed for effective endoscopic suturing, may explain the lower workload associated with this system despite its mechanism being new to users compared to the well-known predicate device. This was particularly evident in challenging locations such as the gastric fundus and the rectum. Additionally, the novel system is compatible with a single-channel endoscope. We anticipate that these will translate into a shorter learning curve resulting in widespread adoption of endoscopic suturing.

This novel test suturing system will require further evaluation in humans. However, results from this porcine study demonstrate a favorable safety profile with effective and efficient defect closure, including full-thickness perforation closure. The use of this device for other indications such as endobariatric procedures will require further evaluation. The sample size in this pilot porcine study reduced statistical power. However, the comparable procedure times, success rates, and lower task loads suggest a favorable safety and efficacy of the test system.

In conclusion, in a porcine model, a novel, FDA-cleared, simple suturing system demonstrated comparable safety and performance to a commercially available predicate suturing platform. The simplified design of the device significantly reduced endoscopist workload, which may offer clinical advantages for routine and complex endoscopic defect closure. Further studies in humans are warranted prior to widespread clinical adoption.

## Supplementary Information

Below is the link to the electronic supplementary material.

Supplementary file1 (DOCX 20 kb)

Supplementary file2 (MP4 138362 kb)

Supplementary file3 (MP4 207959 kb)
