# Supplementary material for: Evaluation of the envision endoscopy SimpleStitch suturing system for closure of gastrointestinal defects in a porcine model
Source: Surg Endosc. 2026 Mar 10;40(4):3510–8. doi: 10.1007/s00464-026-12718-4 (PMC13053462; doi:10.1007/s00464-026-12718-4)
Supplement: Supplementary file 1 — Supplementary file1 (DOCX 20 kb) [file 464_2026_12718_MOESM1_ESM.docx]

**Supplementary tables**

# **Supplementary Table 1: Defect location and device assignment**

|  |  | **Pig no 1** | **Pig no 2** | **Pig no 3** | **Pig no 4** |
| --- | --- | --- | --- | --- | --- |
| **Organ** | **Site location** | **Closure Device** | **Closure Device** | **Closure Device** | **Closure Device** |
| Stomach | Antrum-Anterior | SimpleStitch | SimpleStitch | OverStitch | SimpleStitch |
| Stomach | Antrum-Posterior | SimpleStitch | OverStitch | SimpleStitch | OverStitch |
| Stomach | Body-Anterior | SimpleStitch | SimpleStitch | SimpleStitch | OverStitch |
| Stomach | Body-Posterior | OverStitch | SimpleStitch | SimpleStitch | SimpleStitch |
| Stomach | Fundus-Anterior | SimpleStitch | SimpleStitch | OverStitch | SimpleStitch |
| Stomach | Fundus-Posterior | OverStitch | OverStitch | SimpleStitch | SimpleStitch |
| Colon | Colon-Anterior | SimpleStitch | OverStitch | OverStitch | OverStitch |
| Colon | Colon-Posterior | OverStitch | SimpleStitch | SimpleStitch | SimpleStitch |
| Colon | Rectum-Anterior | SimpleStitch | OverStitch | OverStitch | OverStitch |
| Colon | Rectum-Posterior | OverStitch | SimpleStitch | SimpleStitch | SimpleStitch |

**Supplementary Table 2: Mayo Developmental Endoscopy Unit Score for Mucosal Resection Bed Healing Score**

| **Healing Grade** | **Description** |
| --- | --- |
| IA | Transmural perforation |
| IB | Muscle injury evident |
| IIA | Active bleeding |
| IIB | Bleeding stigmata (visible vessel, adherent clot) |
| III | Clean based ulcer |
| IV | No mucosal defect (healed ulcer) |

# **Supplementary Table 3:Overall Inflammation by Intestinal Layer. The amount of overall inflammation was graded semi-quantitatively for each device implantation site as described in the narrative. Data represent the group mean ± standard deviation (SD).**

| **Device** | **Mucosa/ Submucosa** | **Muscularis** | **Serosa** |
| --- | --- | --- | --- |
| **Test** | 0.8 (0.9) | 1.6 (1.1) | 0.1 (0.3) |
| **Control** | 0.9 (1.2) | 1.5 (0.7) | 0.3 (0.6) |
